# Supplementary material for: Odor-dependent temporal dynamics in Caenorhabitis elegans adaptation and aversive learning behavior
Source: PeerJ. 2018 Jun 12;6:e4956. doi: 10.7717/peerj.4956 (PMC6003392; doi:10.7717/peerj.4956)
Supplement: Figure S1 — Number refers to attraction index (AI), percent refers to the ratio between AI in either ceh-36 or odr-7 mutant animals and the AI in wild-type animals. Error indicate standard error. [file peerj-06-4956-s001.pdf]

odor	wild-type	*ceh-36*	*odr-7*	*ceh-36*/WT	*odr-7*/WT
diacetyl	0.90 ± 0.03	0.83 ± 0.04	0.26 ± 0.02	92.64%	28.96%
benzaldehyde	0.89 ± 0.02	0.03 ± 0.05	0.88 ± 0.04	2.78%	97.38%
butanone	0.84 ± 0.03	0.16 ± 0.06	0.84 ± 0.03	18.40%	98.89%
isobutyric acid	0.71 ± 0.07	0.54 ± 0.07	0.14 ± 0.06	76.66%	19.04%
2-isobutylthiazole	0.87 ± 0.03	0.11 ± 0.16	0.75 ± 0.15	12.28%	85.52%
dimethylthiazole	0.92 ± 0.03	0.25 ± 0.15	0.79 ± 0.11	27.21%	85.56%
2,4,5-trimethylthiazole	0.91 ± 0.03	0.46 ± 0.12	0.74 ± 0.11	50.23%	81.79%
2-methylpyrazine	0.75 ± 0.05	-0.01 ± 0.16	0.59 ± 0.14	-0.98%	78.58%
2-heptanone	0.78 ± 0.03	0.10 ± 0.08	0.79 ± 0.04	12.58%	100.80%
1-methylpyrrole	0.80 ± 0.03	0.10 ± 0.07	0.68 ± 0.05	12.53%	85.10%
4-chlorobenzyl mercaptan	0.73 ± 0.09	0.05 ± 0.14	0.58 ± 0.17	6.14%	79.60%
butyric acid	0.55 ± 0.09	0.36 ± 0.12	0.22 ± 0.07	64.05%	40.41%
1-pentanol	0.77 ± 0.05	-0.09 ± 0.07	0.61 ± 0.07	-11.48%	79.05%
benzyl mercaptan	0.76 ± 0.04	-0.01 ± 0.08	0.54 ± 0.11	-1.20%	71.28%
2-cyclohexylethanol	0.81 ± 0.05	0.00 ± 0.06	0.60 ± 0.12	0.35%	73.46%
2-ethoxythiazole	0.81 ± 0.06	0.10 ± 0.10	0.77 ± 0.05	12.32%	94.83%
benzyl proprionate	0.78 ± 0.05	0.33 ± 0.13	0.27 ± 0.11	42.25%	33.93%
