# Supplementary figures and images for: Odor-dependent temporal dynamics in Caenorhabitis elegans adaptation and aversive learning behavior
Source: PeerJ. 2018 Jun 12;6:e4956. doi: 10.7717/peerj.4956 (PMC6003392; doi:10.7717/peerj.4956)

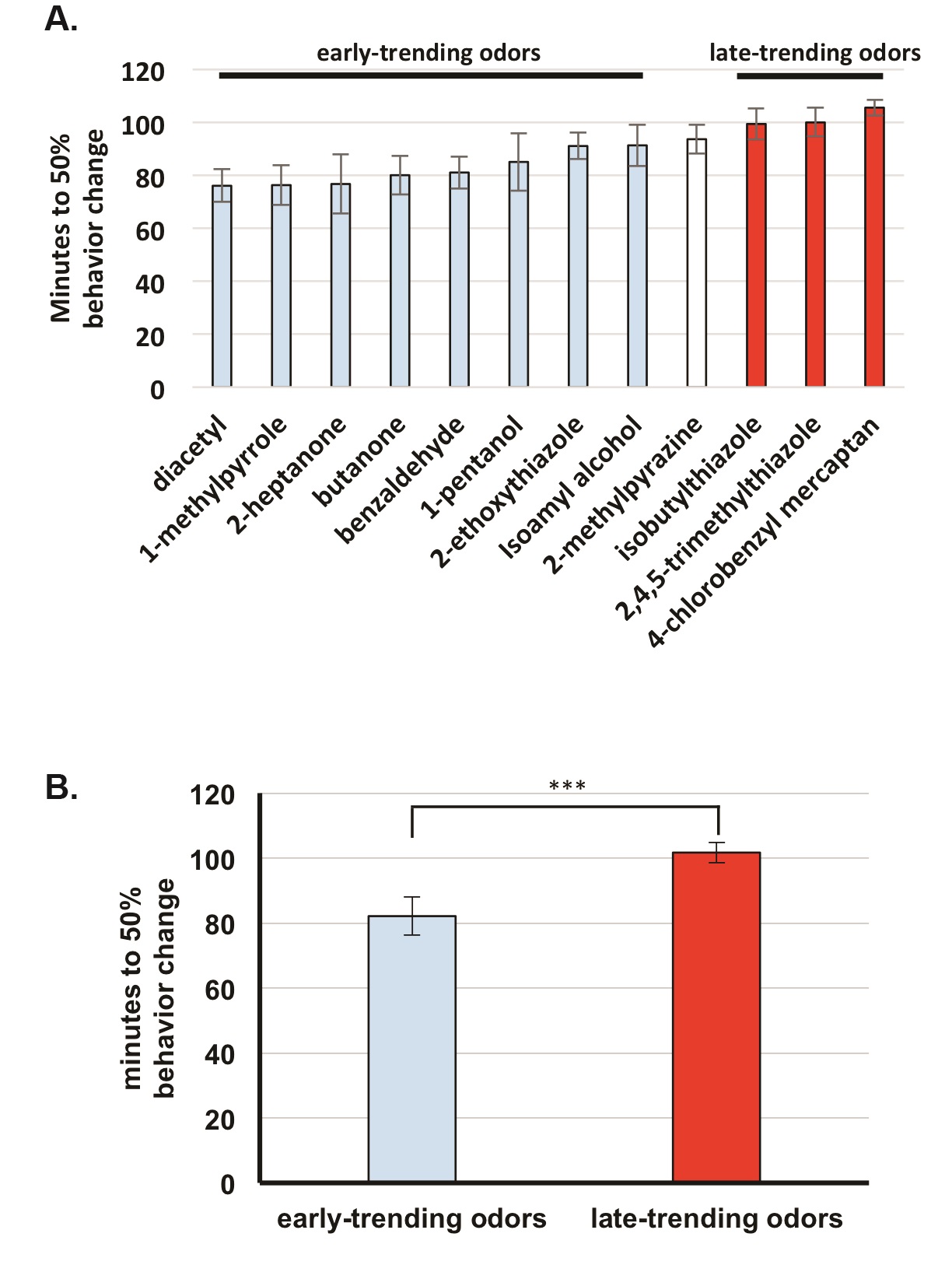

Supplement: Figure S2 — (A) Minutes to 50% change in odor behavior in the real-time behavior assay to 12 odors. Odors were divided into 8 early trending odors (blue) and 3 late-trending odors (red). (B) Early-trending odors reach 50% behavior change faster than late-trending odors. The average of 50% behavior change for the 8 early-trending odors and 4 late-trending odors is shown. Error bars indicate standard deviation. Significance was determined by student’s t-test. *** indicates p < 0.001. [file peerj-06-4956-s002.jpg]

### 4-chlorobenzyl mercaptan

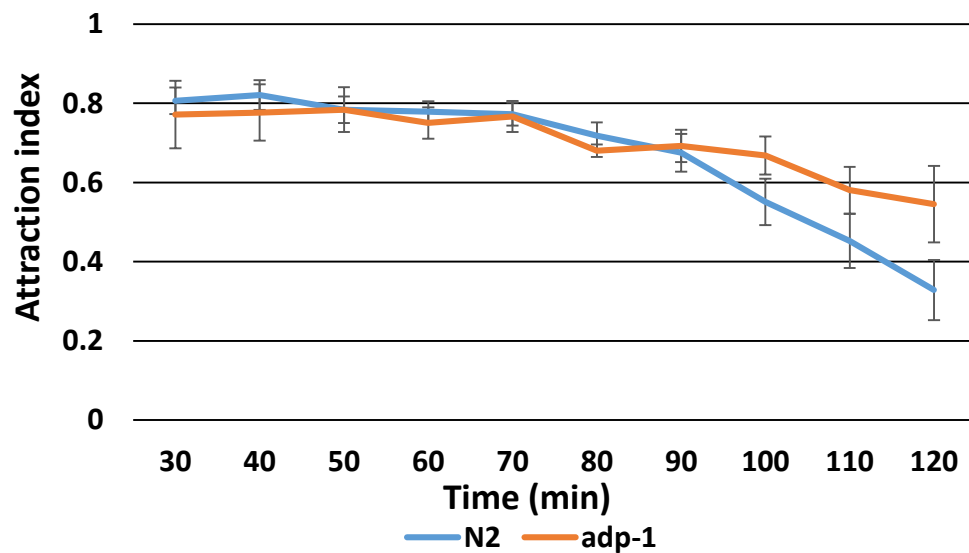

### 2,4,5-trimethylthiazole

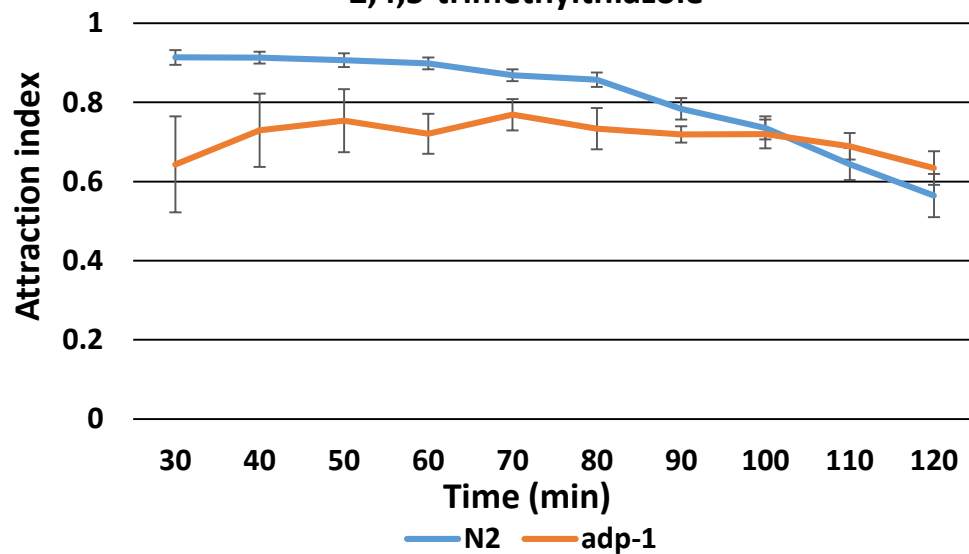

Supplement: Figure S3 — Attraction to 4-chlorobenzyl mercaptan (top) and 2,4,5-trimethylthiazole (bottom) in the real-time behavior assay in wild-type N2 (blue) and adp-1 mutant animals (orange). Error bars indicate standard error. [file peerj-06-4956-s003.pdf]

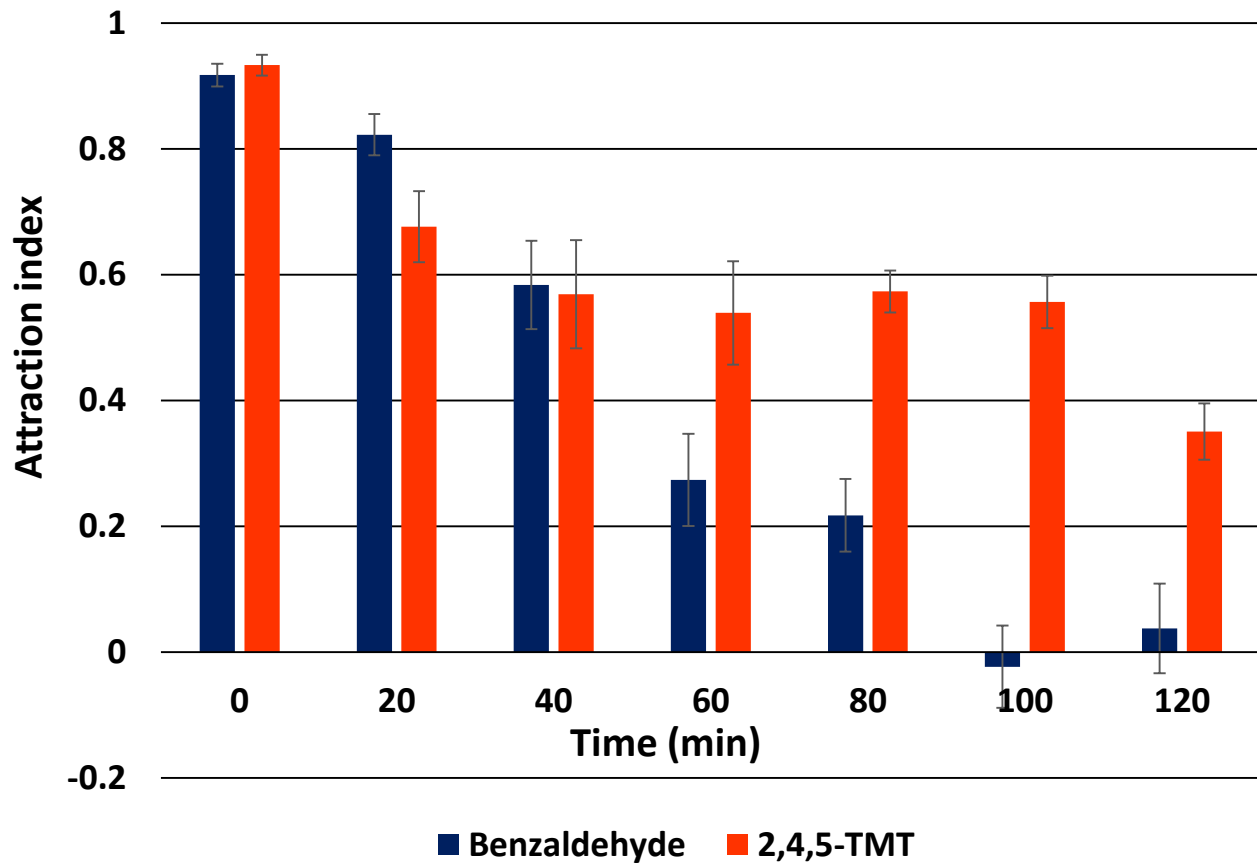

Supplement: Figure S4 — Wild-type N2 animals show early learning to the odor benzaldehyde (dark blue), and late learning to 2,4,5-trimethylthiazole (red). Error bars indicate standard error. [file peerj-06-4956-s004.pdf]

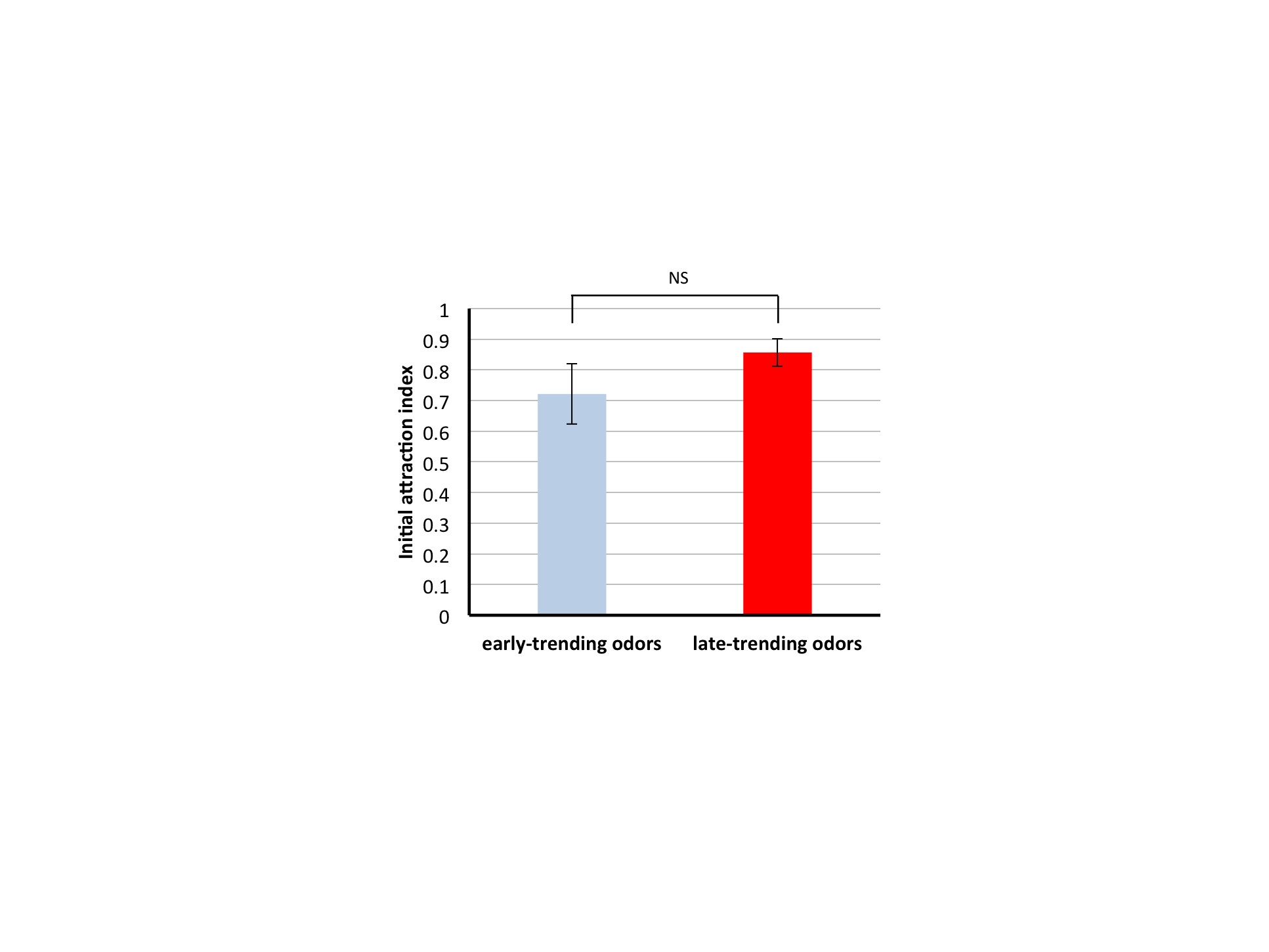

Supplement: Figure S5 — Odor attraction at the beginning of the real-time odor behavior assay (30 minute) was determined for each early-trending odors and late-trending odor and averaged. Error bars indicate standard deviation. NS indicates p > 0.05 by student’s t-test and no statistical significance. [file peerj-06-4956-s005.jpg]

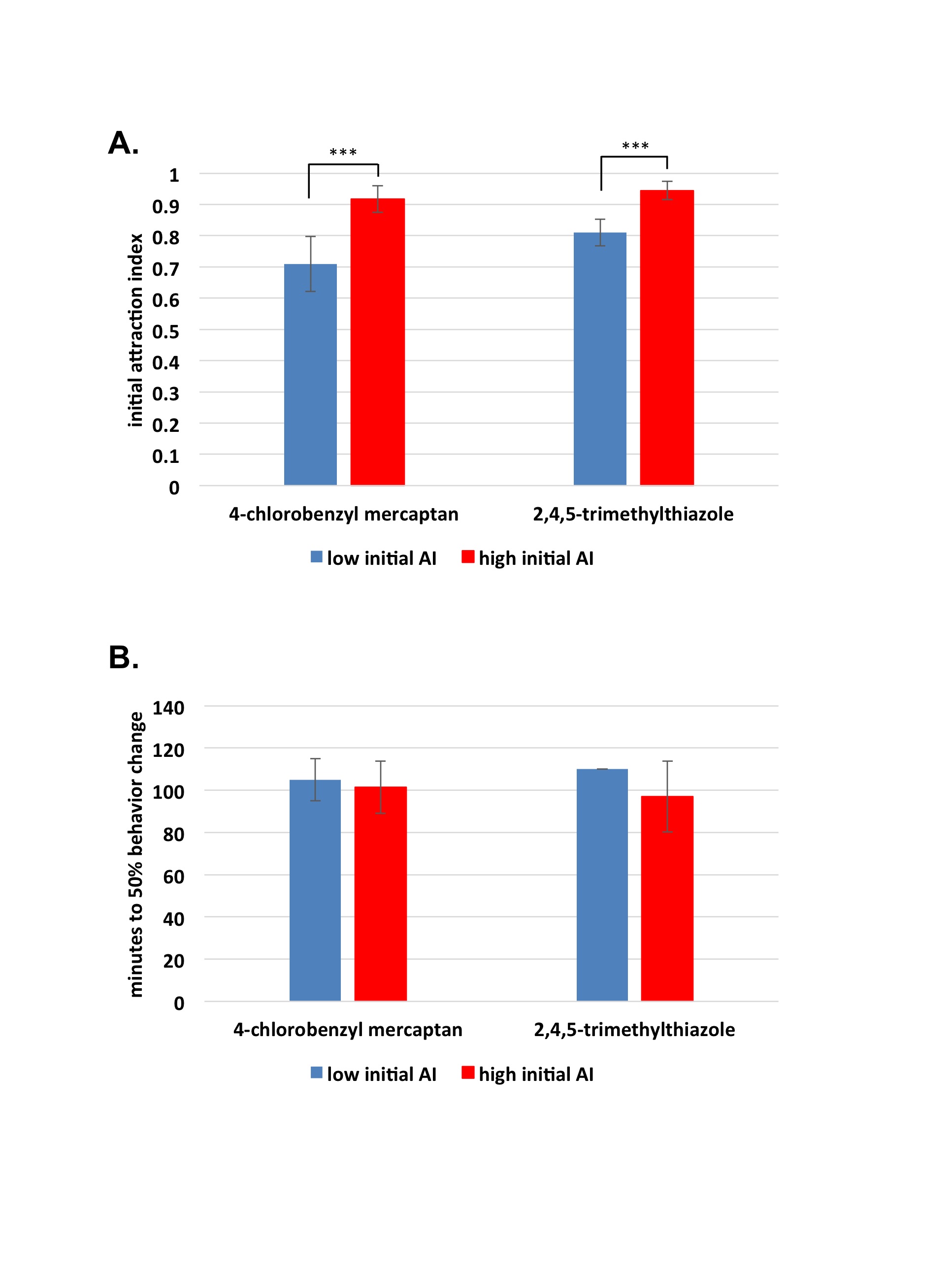

Supplement: Figure S6 — (A) Real-time behavior assay replicated trials towards a 1:100 dilution of 4-chlorobenzyl mercaptan or a 1:1,000 dilution of 2,4,5-trimethylthiazole resulted in varied range of initial AI between trials. These trials were divided into experiments that began with a low initial AI or a high initial AI. (B) Minutes to 50% behavior change for low AI trials and high AI trials towards 4-chlorobenzyl mercaptan and 2,4,5-trimethylthiazole. Low AI trials for either odor did not result in large differences in the timing of behavior change compared to high AI trials. Error bars indicate standard deviation. Significance was determined by student’s t-test. *** indicates p < 0.001. [file peerj-06-4956-s006.jpg]

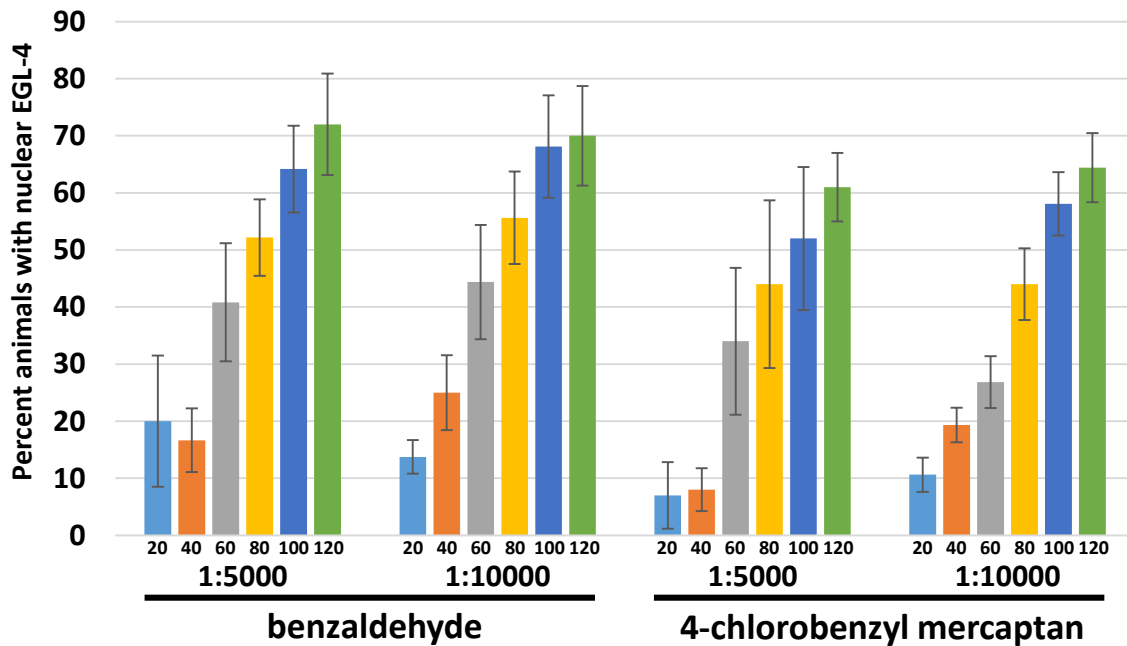

Supplement: Figure S7 — Animals were exposed to two dilutions of the odors and nuclear localization of EGL-4 was assessed in 20 min intervals. Error bars indicate standard error. [file peerj-06-4956-s007.pdf]

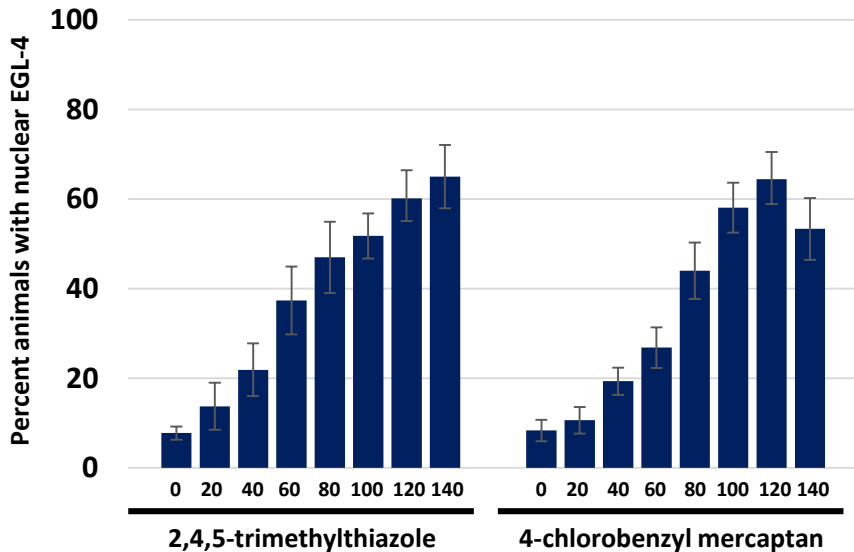

Supplement: Figure S8 — Error bars indicate standard error. [file peerj-06-4956-s008.pdf]
